# Supplementary material for: Observational study of factors associated with morbidity and mortality from COVID-19 in Lebanon, 2020–2021
Source: PLoS One. 2022 Oct 19;17(10):e0275101. doi: 10.1371/journal.pone.0275101 (PMC9581355; doi:10.1371/journal.pone.0275101)
Supplement: S1 Table — (DOCX) [file pone.0275101.s001.docx]

**Table S1**. Categorical and quantitative variable definitions

| **Variable** | **Definition** | **Based on** |
| --- | --- | --- |
| PlateletsC | 150-400, Scaled and centered | Platelet count (10^3/¬µl) |
| VolumeC | Scaled and centered | Max vol of oxygen (L) needed during stay |
| CRPC | Normal <10, Scaled and centered | CRP (mg/l) |
| HbC | Normal 13.8-17.2 (men), 12.1-15.1 (women), Scaled and centered | Hb (g/dL) |
| AgeC | 1 if age ≥65, else 0 | Age |
| WBCC | WBC ≥ 11 | WBC (10^3/¬µl) |
| CreatinineC | Creatinine > 1 for women, > 1.2 for men | Creatinine (md/dl) |
| SGOTC | SGOT (AST) ≥43 for women, 48 for men | SGOT (AST) (IU/L) |
| SGPTC | SGPT (ALT) ≥45 for women, 55 for men | SGPT (ALT) (IU/L) |
| SexC | Sex - 1; Sex = 2 for women, 1 for men | Sex |
| DDimerC | ≥ 250 | D Dimer (ng/ml) |
| Ferritin | ≥ 160 women, ≥300 for men | Ferritin (ng/ml) |
| Obesity | >30 | Body Mass Index |
