# Supplementary material for: Observational study of factors associated with morbidity and mortality from COVID-19 in Lebanon, 2020–2021
Source: PLoS One. 2022 Oct 19;17(10):e0275101. doi: 10.1371/journal.pone.0275101 (PMC9581355; doi:10.1371/journal.pone.0275101)
Supplement: Attachment — Submitted filename: Rebuttal-PZ-02.09.2022.docx [file pone.0275101.s005.docx]

September 2^nd^, 2022

To Eili Y. Klein, PhD

Academic Editor
PLOS ONE

Dear Professor Klein,

We thank the reviewer for agreeing with the changes made to the revised manuscript which undoubtedly made it more valuable. We very much appreciate the editor’s valuable input and careful assessment (and edits) to further improve our manuscript.

We have now addressed all the comments and implemented the requested changes as evidenced in the Track Change version of the revised document.

Please find below a point-by-point rebuttal to the editor’s comments. We hope that our revised manuscript is now acceptable for publication at PLOS One.

Pierre Zalloua,

On behalf of all Authors.

Editor’s comments:

The methods suggest that multivariate logistic regressions were done, yet I only see binomial regression results. This may just be mislabeled - but needs to be clarified in line with publication criteria #3. I would highly recommend that the authors follow the STROBE guidelines for reporting on observational studies (<https://www.ncbi.nlm.nih.gov/pmc/articles/PMC2870880/>), that will ensure that the results are clear. Finally, the title suggests just creatinine levels were important, but that is only part of the story as creatinine was highly associated with hypertension, and it probably is beyond the capability of this study to disentangle the relationship. I would suggest that the title more accurately reflect the study type (as per STROBE guidelines) as well as more accurately reflect results. For instance Observational study of factors associated with Morbidity and Mortality from COVID-19 in Lebanon, 2020-2021 or something similar.

We thank the editor for pointing out this matter and we regret the confusion.

We used the term multivariate logistic regressions since our linear models included multiple factors, such as adjustment variables, and we did not state that we performed multinomial tests. We realize that this terminology is confusing since our multivariate regression was in fact a simple binomial logistic regression (in the tables we used that term). We have now removed the term multivariate, and we used logistic regression throughput. We kept the label binomial in the Tables’ captions. We also followed the STROBE guidelines as suggested. We addressed all other comments raised in the revised version of the manuscript and these include a change of the title, and tables as suggested.

Minor comments

1. Add timeframe to methods

We added the timeframe in the methods section of the Abstract.

2. Remove use of correlation where not appropriate

We inadvertently missed the removal of correlate/correlation from the previous submission although we have indicated this change in the rebuttal. We thank the editor for pointing out to this input error. We have now removed correlate/correlation from the revised manuscript.

3. Reduce the introduction length by removing extraneous passages and unlinked information

We revised the introduction, and we removed extraneous passages and unlinked information.

4. Please ensure the correct tense is used throughout

We ensured that the correct tense is used throughout.

5. Be more clear about the other biomarkers and why only creatinine is your focus given that it was so highly correlated with hypertension

We clarified this aspect in the discussion, and we shed light on the interaction between creatinine and hypertension.
